# Supplementary material for: FarmCPUpp: Efficient large‐scale genomewide association studies
Source: Plant Direct. 2018 Apr 10;2(4):e00053. doi: 10.1002/pld3.53 (PMC6508500; doi:10.1002/pld3.53)
Supplement: Supplementary file 1 [file PLD3-2-e00053-s001.pdf]

Reviewer Comments:
Reviewer #1:
Comments to the Author (Required):
I think that this paper does a great job of describing the differences between FarmCPU and FarmCPUpp, and the simulation studies and real data analysis effectively demonstrate the reduction in computational time. I have only one major question/suggestion: why was the maize NAM population used as a test data set? The reason I ask is that FarmCPU is most often used to analyze diversity panels and was developed (as an alternative of the unified mixed linear model) to adjust for sources of false positives that are commonly found in diversity panels. Therefore, I wonder if it would be more effective to analyze some real data from a diversity panel (e.g., the maize NCRPIS aka "Ames" panel) instead of the NAM population.

Minor editorial suggestion: for the paragraph in lines 33-36: I strongly suggest acknowledging GEMMA in this paragraph. Indeed, GEMMA is the "best of both worlds" in the sense that it is as computationally efficient as, say, EMMAX/P3D, but one can actually get exact parameter estimates. Here is the citation for GEMMA:

Zhou X, Stephens M: Genome-wide efficient mixed-model analysis for association studies. Nat Genet 2012, 44:821 U136

Reviewer #2:
Comments to the Author (Required):
FarmCPUpp addresses many of the major usability issues with the initial implementation of the FarmCPU algorithm. I have installed and run FarmCPUpp successfully and I get the expected high confidence results. I appreciate the thorough review of other advances in GWAS analysis and the thorough comparison to the existing FarmCPU package.

Transferred Reviewer Comments:
